# Tribological Properties of Oil-in-Water Emulsion with Carbon Nanocapsule Additives

**DOI:** 10.3390/ma13245762

**Published:** 2020-12-17

**Authors:** Yeau-Ren Jeng, Ping-Chi Tsai, Ching-Min Chang, Kuo-Feng Hsu

**Affiliations:** 1 Department of Biomedical Engineering, National Cheng Kung University, Tainan 701401, Taiwan; pctjbenchen@gmail.com (P.-C.T.); jimi690504@gmail.com (C.-M.C.); 2 Department of Mechanical Engineering, National Chung Cheng University, Chia-Yi 621301, Taiwan; az123123@livemail.tw

**Keywords:** carbon nanocapsule additives, water-based lubricant, emulsified oil, tribological performances

## Abstract

An experimental investigation was performed on the coefficients of friction (COFs) and wear properties of pure water and oil-in-water (O/W) working fluids containing carbon nanocapsules (CNCs) with concentrations ranging from 0 to 1.0 wt.%. For the O/W working fluid, the ratio of oil to water was set as 6%. It was shown that for the water working fluid, the COF decreased by around 20% as the CNC content increased from 0 to 1.0 wt.%. In contrast, the wear volume increased by 50% as the CNC addition increased from 0 to 0.5 wt.%, but it fell to a value slightly lower than that achieved using only pure water (i.e., no CNCs) as the CNC content was further increased to 1.0 wt.%. For the O/W emulsion, the addition of 0.8 wt.% CNCs reduced the COF by around 30% compared to that of the emulsion with no CNCs. Overall, the results showed that while the addition of a small quantity (6%) of oil to the water working fluid had a relatively small effect on the wear performance, the addition of an appropriate quantity of CNCs (i.e., 0.8 wt.%) resulted in a significantly lower COF and an improved wear surface.

## 1. Introduction

As the demands placed on modern industry for greater machining precision, faster throughputs, and improved quality continue to increase, the efficiency, speed, and accuracy of the machining systems used in the production process are becoming increasingly more important. The problems of friction, wear, and lubrication have, thus, attracted great interest in recent years [1,2]. Water-based emulsions (oil-in-water (O/W)) are widely used to improve heat dissipation performance and reduce friction and wear in metal working processes such as cutting and rolling [3,4,5]. However, traditional emulsions typically contain elements such as phosphorus, sulfur, and chlorine that are not environmentally friendly. Consequently, disposing/recycling of the waste emulsion following the machining process is a time-consuming and expensive process. The problem of replacing these additives with more environmentally sustainable alternatives has, therefore, emerged as a major concern. Among the various additives that have been proposed, nanoparticles have attracted particular attention because, compared to traditional additives, they undergo only limited chemical reaction under the effects of friction heating [6]. Moreover, nanoparticles have a natural filling capability and are, therefore, an ideal material for the repair of worn surfaces [7,8,9].

Many metallic nanoparticles have been proposed for improving the lubrication performance of water working fluids, including titanium dioxide (TiO_2_), silicon dioxide (SiO_2_), zirconium dioxide (ZrO_2_), zinc oxide (ZnO), and graphite. Gao et al. [10] showed that the addition of 0.5 wt.% oleic acid-modified TiO_2_ nanoparticles to water greatly reduced the abrasive effect at the contact surface. Similarly, Wu et al. [11] showed that the coefficient of friction (COF) and wear characteristics of water lubricant on ferritic stainless steel were both significantly improved through the addition of 4 wt.% TiO_2_ nanoparticles. Dinget al. [12] showed that the addition of 5 wt.% amino-modified SiO_2_ nanoparticles to water lubricant reduced the COF by 38.3%. Zhong et al. [13] added short carbon fibers (SCFs) and ZrO_2_ nanoparticles to water lubricants and showed that the composite particles yielded a significant reduction in wear resistance at the contact surface, particularly under high pressures. Gara and Zou [14] investigated the friction and wear characteristics of ZnO and aluminum oxide (Al_2_O_3_) nano-additive water-based lubricants with varying concentrations on different rough surfaces. The results showed that addition of ZnO nanoparticles to deionized water reduced friction by up to 56.9% as the concentration increased. Al_2_O_3_ nanofluids also reduced friction, although not as much as ZnO nanofluids. Zhang et al. [15,16] examined the tribological properties of as-prepared nano-Cu and Cu/SiO_2_ nanocomposites in distilled water. It was shown that the two additives resulted in effective improvement of tribological performance as a result of the formation of a boundary lubrication film and a protective film at the contact surface, respectively.

In addition to the nanoparticles described above, the use of nanoparticles containing carbon as lubricant additives in water has also attracted increasing attention in recent years due to their unique combination of properties, including low friction and high hardness. Various carbon-containing nanoparticles have been proposed, including carbon nanotubes (CNTs), graphene, graphite alkyne, diamond, and carbon black [17,18,19,20,21,22,23,24]. Peng et al. [18] showed that the addition of SDS-functionalized multi-walled carbon nanotubes (MWCNTs) to water yielded an effective reduction in the friction coefficient and wear rate of friction pairs in a steel–steel sliding system. Similarly, Kinoshita et al. [19] reported that graphene oxide (GO) nanosheets readily attached to friction surfaces in water and formed a protective coating that significantly improved the friction-reducing and anti-wear properties of the surface. Elomaa et al. [20] also showed that GO nanosheets readily adhered to diamond-like carbon and stainless-steel surfaces and, therefore, improved the COF and wear resistance properties of water-based lubricants by increasing the number of water molecules adsorbed by the contact surface.

The literature also contains several studies on the effectiveness of nanoparticles in enhancing the tribological performance of emulsions [25,26,27,28]. These studies generally considered GO nanoparticles as the additive, and they focused primarily on the effects of the structure and size of the particles on the lubrication performance of the emulsion. However, chemically synthesizing GO nanoparticles involves a long and complex procedure [29]. In comparison, carbon nanocapsules (CNCs), with a more cost-effective and scalable synthesis process, are regarded to be a more attractive option for tribological applications [30,31]. In a previous study [30], we added CNCs with various concentrations to mineral oil lubricant and evaluated the friction characteristics and wear behavior at the contact interface using block-on-ring tests. The results showed that CNCs led to a significant reduction in the COF at the contact surface due to their structural evolution under the effects of the contact force.

However, none of the studies described above considered the use of surfactants to improve the dispersion of nanoparticles in the working fluid. Accordingly, the present study prepared two working fluids consisting of pure water and O/W emulsion, respectively, containing CNCs with concentrations ranging from 0 to 1.0 wt.%. The uniformity of the CNCs dispersion in the former working fluid was enhanced by the addition of 2% SL 70 surfactant (Nouryon, Amsterdam, Netherlands) [32], and that in the latter working fluid was further improved by adding an oil phase to the water-based emulsion, in which the SL 70 surfactant was used to reduce the surface tension between water and oil molecules to form a kinetically stable emulsion.

The lubrication performance of the two working fluids was investigated by sliding tests performed using block-on-ring wear tests with SKD11 rings and blocks. Following the wear tests, the tribological performance of the two working fluids was evaluated using a surface profilometer (SMS-300A, BMT, Ettlingen, Germany) and scanning electron microscopy (SEM, S-4800, Hitachi, Kagoshima, Japan).

## 2. Experimental Procedure and Methods

### 2.1. CNC Preparation Method

The CNCs were prepared by a pulse plasma arc discharge method using argon gas as the working fluid. Briefly, argon gas (maintained at a pressure of 1–2 atm) was flowed into a chamber containing a graphitic cathode and anode. A DC pulse current (60 Hz; −20 V; −120 A) was then applied to one of the electrodes to initiate a carbon arc reaction. After 30 min, the deposit formed on the cathode was collected and suspended in water by surfactant encapsulation. It was then passed through a 0.22 mm filter to separate the CNCs and carbon nanotubes. Finally, the surfactant was removed from the CNCs using high-speed centrifugation.

The CNCs were added to the water and O/W emulsion at concentrations of 0–1.0 wt.%. Prior to mixing with the lubricants, the CNCs were surface modified via hydroxylation. As described in previous studies [30,31], the hydroxyl radical is chemically reactive toward the C=C double bonds of the CNCs and, thus, prompts the formation of a hydroxyl group covalently bound to the CNCs surface via free-radical addition. Following the hydroxylation modification process, the CNCs formed a black homogeneous solution in water and were then added to 6% oil to formulate the water-based emulsion. As the percentage increases further, the stability of the emulsion may decrease due to rapid coalescence of oil droplets [33,34].

**Figure 1:**
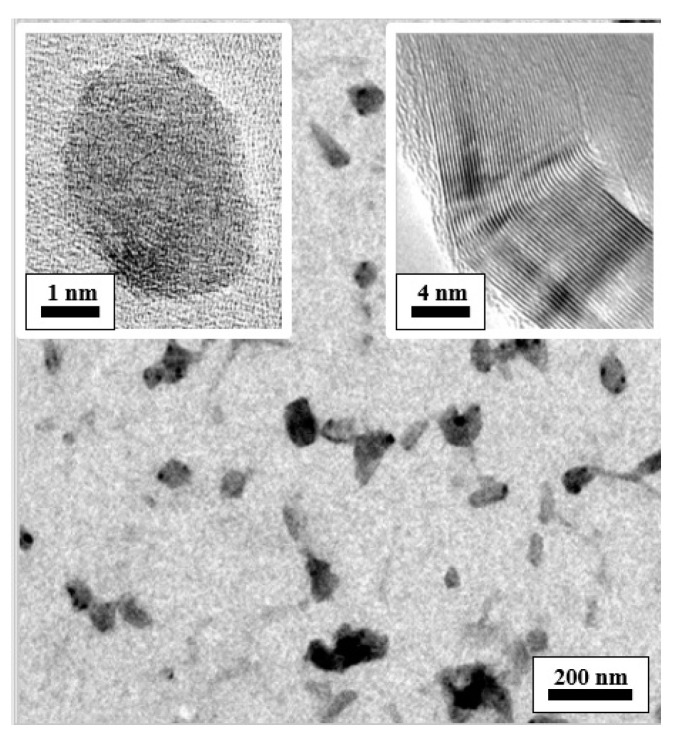
TEM images of carbon nanocapsules (CNCs) dispersed in water.

**Figure 2:**
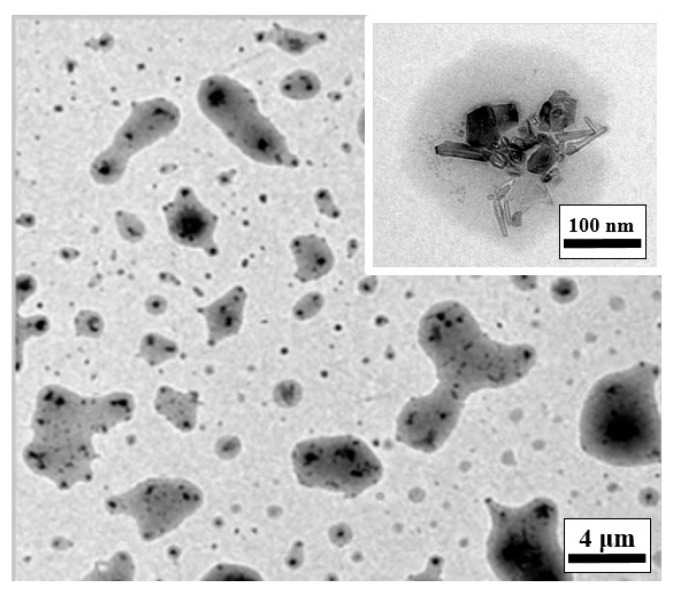
TEM images of CNCs dispersed in emulsified oil.

Figure 1 and Figure 2 present transmission electron microscope (TEM, JEM-2010, JEOL, Tokyo, Japan) images of the CNCs dispersed in water and emulsified oil, respectively. It is apparent that the chemical functionalization process resulted in no noticeable damage to either the inner graphene layers of the CNCs or their overall integrity.

### 2.2. Evaluation of Tribological Performance of CNCs Working Fluids

**Figure 3:**
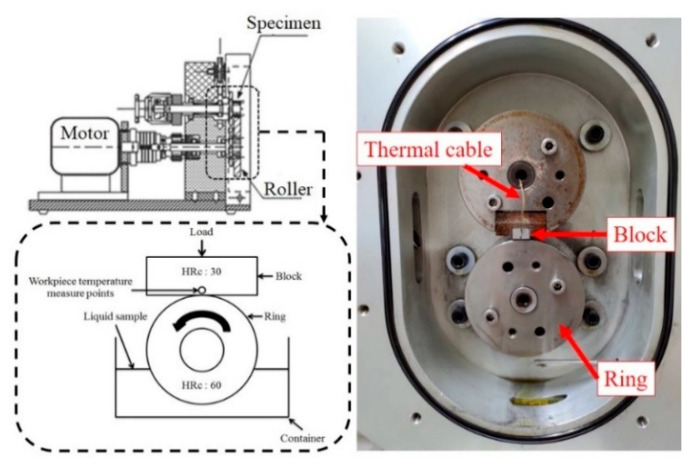
Schematic diagram of block-on-ring arrangement used in the friction and wear experiments.

The tribological properties of the various lubricants were evaluated using block-on-ring wear tests with the configuration shown in Figure 3. The blocks and rings were cleaned ultrasonically in water, wiped with ethanol (Delight, Taoyuan, Taiwan (R.O.C.)), and then dried in air prior to assembly in the machine. In performing the tests, a slider lead screw mechanism was used to move the block in the downward direction to achieve the desired contact force, and a motor was employed to rotate the ring-shaped test piece such that a relative motion was produced between the ring and the block. As shown in Figure 3, the lower part of the rotating test piece was immersed in the working fluid such that the contact surface between the ring and the block was continuously lubricated throughout the wear test. Moreover, the load acting at the contact interface in the X- and Z-directions was measured using a force sensor (DFH-20-G, Bruker Taiwan, Hsinchu, Taiwan (R.O.C.)) attached to the block.

The block and the ring used in the tribological tests were fabricated from SKD11 cold-work tool steel (JFS, Taichung, Taiwan) with Rockwell hardness values of around 28–33 HRC (Unit of Rockwell hardness) and 58–62 HRC, respectively, in which the element compositions of SKD11 materials are shown in Table 1. Table 1 shows that the SKD11 workpiece was well-controlled, with a relatively low content of chemical compositions, implying that the influences of other chemical compositions originating from SKD11 on the following friction and wear experiments can also be reasonably ignored. For the experiments performed using the water working fluid, the load acting on the block was set as 80 N, while the rotation speed was set as 250 rpm. In addition, the test time was set as 40 min. The relative sliding speed was calculated to be 0.458 m/s, while the total sliding distance and Hertz pressure were 1100 m and 49.7 MPa, respectively. For the O/W lubricant, the oil improved the lubrication effect to better observe the effect of friction, and the load was increased to 120 N. In addition, the total sliding time was increased to 60 min, and the rotation speed was maintained at 250 rpm; hence, the total sliding distance was equal to 1375 m, while the Hertz pressure was 60.9 MPa. The experimental parameters used in the various wear tests are summarized in Table 2. In order to isolate the tribofilm effect on friction and wear between pure water and emulsion, the specific conditions presented in Table 2 were prepared to minimize inherent errors arising from tribofilm formation.

For both lubricants, the COF was measured under steady-state conditions during the experiment. Note that a typical calibration procedure and two-sigma rule (i.e., 95% within two standard deviations technique) was performed to better interpret the experimental data from friction and wear tests. A similar calibration procedure and standard deviation technique were used to estimate experimental errors in the mechanical and tribological responses of carbon-based composites [35,36]. All tests were carefully performed under the chosen conditions. However, there were still uncontrolled factors due to the non-uniform sized and unstable distribution of CNCs. However, obtaining a well-dispersed condition for water-based working fluid has been an ongoing challenge, and the size of nanoparticles are known to have a range of distribution.

### 2.3. Wear Property Measurement and Abrasion Evaluation

Following the wear tests, the surface roughness and wear morphologies of the workpieces were measured using a surface profilometer and SEM. For each test piece, the surface height was measured using a surface profilometer with a resolution of 300 points/mm. The wear volume was then estimated classically using pore volume analysis techniques integrated with the measurement system. The abrasion marks on the wear surfaces were additionally observed through SEM to qualitatively determine the effects of different CNC concentrations on the wear performance of the two lubricants.

## 3. Results and Discussion

### 3.1. Surfactant-Enhanced Dispersion of CNCs in Water Lubricant

For lubricants containing nanoparticles (i.e., CNCs), it is essential that the particles are evenly suspended in the solution to minimize the COF and achieve consistent wear performance over the contact area [30,31]. Table 3 summarizes the results obtained from a preliminary series of experiments designed to investigate the effectiveness of various commercial surfactants in dispersing the prepared CNCs within the water lubricant. As shown, the SL 70 surfactant enabled up to 1 wt.% of CNCs to be evenly dispersed. In contrast, the SDS and CAS surfactants enabled the dispersion of 0.4 wt.% CNCs, while the surfactant cetyltrimethylammonium bromide (CTAB) enabled the dispersion of only 0.3 wt.% CNCs. As such, SL 70 was chosen as the surfactant in all of the remaining experiments [32].

**Figure 4:**
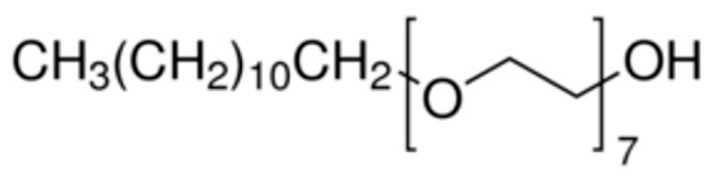
Heptaethylene glycol monododecyl ether, SL 70 (C_12_E_7_).

SL 70 is a non-ionic surfactant. As shown in Figure 4, the ether end (on the left) has polarity and serves as the hydrophilic end, while the primary carbon atom on the right end is non-polar and serves as the hydrophobic end. In other words, the primary carbon atom combines with the CNC additives, while the polar molecules on the ether end combine with water molecules.

### 3.2. Friction Coefficients of Water Lubricant with Different CNC Concentrations

**Figure 5:**
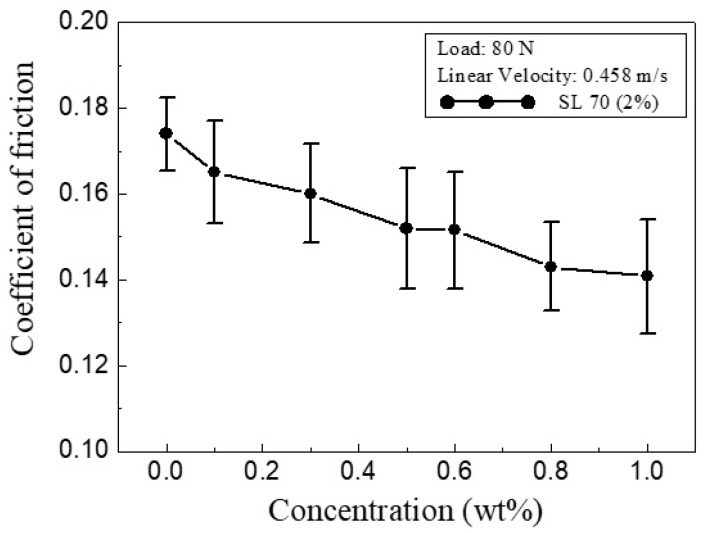
Coefficient of friction (COF) variation of different CNC concentrations in water working fluid.

Figure 5 shows the variation of the average COF of the water lubricant with the CNC concentrations under an applied load of 80 N and a sliding velocity of 0.458 m/s. Adding CNCs from 0 to 0.1 wt.% drastically reduced the COF. Further increase in CNCs from 0.1 to 0.8 wt.% led to a steady reduction in friction with increasing CNC concentrations. Notably, the results also suggested that, because the COF declined only very slightly as the CNC concentration increased from 0.8 to 1.0 wt.%, a CNC concentration of 0.8 wt.% represented an effective tradeoff between the tribological performance of the water-based working fluid and cost.

### 3.3. Analysis of Wear Surface for Water Lubricant with Different CNC Concentrations

**Figure 6:**
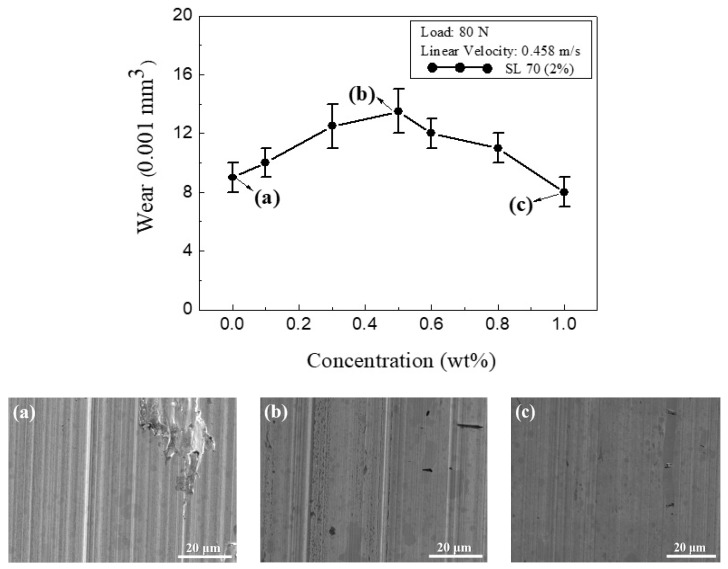
Variation of wear volume with CNC concentration in water working fluid. SEM images show wear surfaces following sliding with water lubricant with (**a**) 0 wt.%, (**b**) 0.5 wt.%, and (**c**) 1.0 wt.% CNC additions.

Figure 6 shows the variation of the wear volume with CNC addition for the water working fluid. For the pure water lubricant with no CNC addition, the wear volume was 0.009 mm^3^. As the CNC concentration increased to 0.5 wt.%, the wear volume increased to 0.0135 mm^3^. As the CNC concentration was further increased to 1.0 wt.%, the wear volume declined to a minimal value of 0.008 mm^3^. Specifically, Figure 6a shows SEM images of the wear surfaces of the test pieces (flat blocks) after the sliding experiments were performed using water lubricants with no CNC particles. The wear surface clearly showed scratch marks and regions of severe gouging. Figure 6b shows the deep grooving and gouging that was reasonably reduced for low-to-medium CNC concentrations, in which the substantial abrasion condition shown in Figure 6b is an indication that the material removal rate reached its maximum value with a CNC addition of 0.5 wt.%. Figure 6c shows a wear surface with polished abrasion conditions. This is likely a result of the greater number of exposed CNCs that may act as an interfacial separator, not only to prevent wear but also to provide a rolling-bearing effect [37,38,39,40]. As such, the lowest friction and fine polishing of rolling abrasion resulted in the minimum wear volume at a CNC addition of 1.0 wt.%.

### 3.4. Friction Coefficients of Different CNC Concentrations in 6% Emulsified Oil

**Figure 7:**
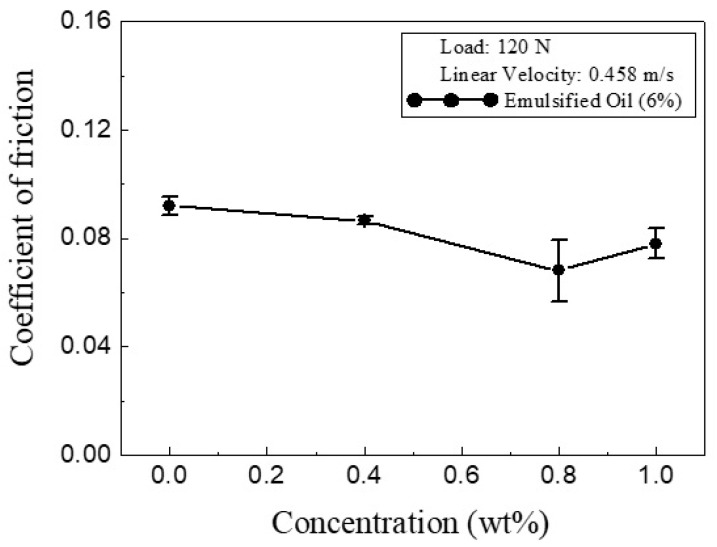
COF variation of different CNC concentrations in 6% emulsified oil.

Figure 7 shows the variation in the average COF of the O/W emulsion with CNC concentrations given a contact load and sliding speed of 120 N and 0.458 m/s, respectively. The ratio of oil to water was set as 6% in every case. Comparing the results presented in Figure 7 with those shown in Figure 5 for the water lubricant, it is apparent that the average COFs of the O/W emulsion were lower than those of the water lubricant, despite the greater magnitude of the contact force. In other words, the addition of a small quantity of oil to the water lubricant was beneficial in reducing the friction force at the contact surface. As the CNC concentration increased from 0 to 0.8 wt.%, the COF of the O/W emulsion reduced to 0.068, representing a significant reduction of around 30%.

Machining processes in industry are commonly performed using emulsified oil lubricants due to their high heat dissipation ability and good rust resistance under high temperatures. However, to achieve this performance, it is generally necessary to maintain the emulsion concentration at around 10% [33,34]. In contrast, in the present study, the emulsion concentration was reduced to only 6% through the addition of a small quantity (less than 1 wt.%) of CNCs. Overall, the results presented in Figure 5 and Figure 7 show that, while the addition of oil to the water working fluid resulted in a relatively slight improvement in the lubrication effect, the further addition of an appropriate quantity of CNCs (i.e., 0.8 wt.%) led to a significant reduction in COF. It should be noted that the emulsified oil with CNCs yielded a lower friction coefficient obtained from block-on-ring tests than that without CNCs, as shown in Figure 7. This can be attributed to the fact that the addition of CNCs resulted in a separation between contact interfaces due to their rolling-bearing effects. A similar observation was reported in a series of previous studies [37,38,39,40] investigating the role of nanoparticles in O/W-based lubricants. In other words, CNCs appear to be an extremely effective additive for emulsified oil lubricants.

### 3.5. Analysis of Wear Surface for Oil/Water Emulsion Lubricant with Different CNC Concentrations

**Figure 8:**
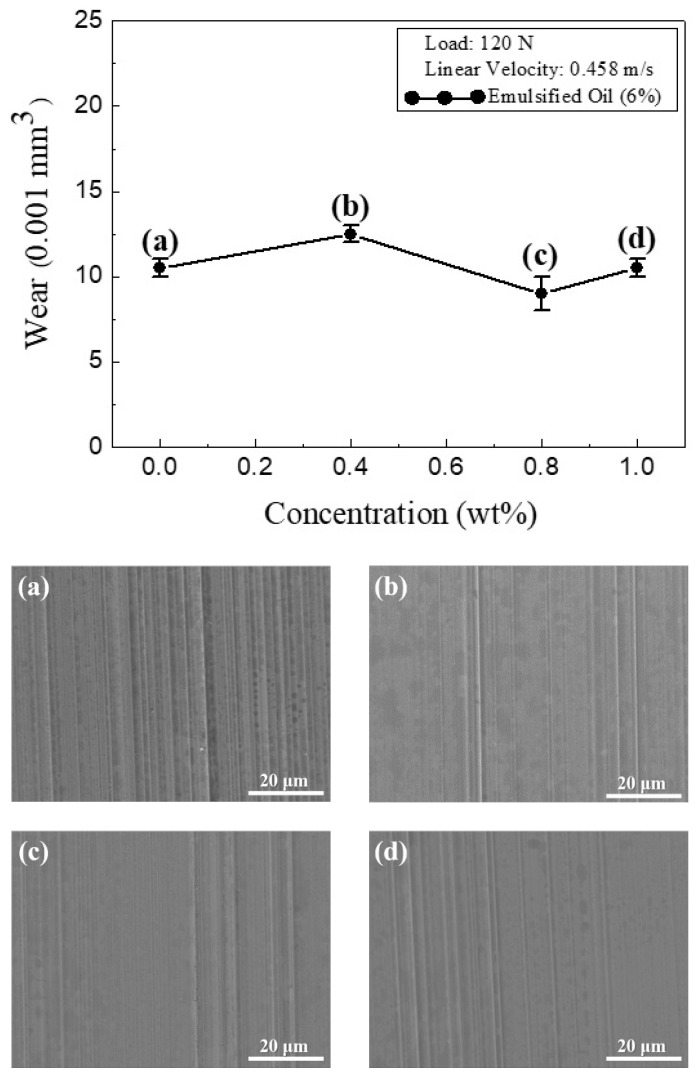
Variation of wear volume with CNC concentrations in O/W emulsions. SEM images show wear surface following sliding with O/W emulsion lubricant with (**a**) 0 wt.%, (**b**) 0.4 wt.%, (**c**) 0.8 wt.%, and (**d**) 1.0 wt.% CNC additions.

Figure 8 shows the variation in wear volume with CNC concentrations in O/W lubricant. As shown, the wear volume varied in a narrow range, from 0.009 to 0.011 mm^3^. Notably, the precision of the surface profilometer was around 0.001 mm^3^. Thus, the results showed that the wear volume varied with increasing CNC concentrations, which were reflected in the SEM images of the wear surfaces produced in the sliding tests performed using the O/W emulsion with CNC concentrations of 0 wt.%, 0.4 wt.%, 0.8 wt.%, and 1.0 wt.%, respectively (Figure 8a–d). The images clearly show that the surfaces of the workpieces processed using lubricants with 0 wt.% and 0.4 wt.% CNCs, respectively, contained more obvious scratch marks than those processed by lubricants with 0.8 wt.% and 1.0 wt.% CNCs. Although Figure 8 shows that CNC concentration had little effect on the material removal rate at the contact surface, the images presented in Figure 8a–d show that the surface morphology became smoother as the CNC concentration increased. This finding most likely reflects a more uniform distribution of CNCs within the O/W emulsions under higher CNC concentrations [41,42]. Overall, the CNC content actually had very little effect on the abrasion performance at the wear surface due to the presence of the oil, which provided a strong lubricating effect.

## 4. Conclusions

Traditional oil emulsions contain elements such as phosphorus, sulfur, and chlorine that are harmful to the environment. Consequently, the problem of developing water-based systems to replace, or at least reduce, the amount of oil in such emulsions has attracted great interest in recent years. This study has, thus, evaluated and compared the tribological performance (COF and wear volume) of two lubricants, namely pure water and O/W emulsion (with the ratio of O/W set to 6%), respectively, containing CNC additives at concentrations of 0 to 1.0 wt.%. The results showed that when using 2% SL 70 surfactant, the COF of the water working fluid reduced by almost 20% as the CNC concentration increased from 0 to 1.0 wt.%. Moreover, the wear volume increased by around 50% as the CNC concentration increased from 0 to 0.5 wt.%, but then it fell to a value slightly lower than that achieved using pure water with no CNC addition as the CNC concentration was further increased to 1.0 wt.%. For the O/W emulsion, the COF decreased by around 30% as the CNC concentration increased to 0.8 wt.%, and then it increased slightly as the CNC concentration was further increased to 1.0 wt.%. The addition of CNCs had no obvious effect on wear volume due to the strong lubricating effect of the oil. However, the wear surface became smoother as the CNC concentration increased because the lower COF reduced the mechanical vibration effect caused by friction. Overall, the results obtained in this study indicate that for both lubricants, a CNC addition of 0.8 wt.% represents a suitable tradeoff between tribological performance of the lubricant and cost.

## Figures and Tables

**Table 1:** Element compositions of SKD11 materials for block-on-ring wear tests.

Element	Content (%)
Carbon (C)	1.5
Silicon (Si)	0.3
Manganese (Mn)	0.45
Chromium (Cr)	12.0
Molybdenum (Mo)	1.0
Vanadium (V)	0.35

**Table 2:** Experimental parameters used in block-on-ring friction and wear test experiments.

Condition	Pure Water	Emulsion
Load (N)	80	120
Rotating speed (rpm)	250	250
Experimental time (min)	40	60
Sliding velocity (m/s)	0.458	0.458
Sliding distance (m)	1100	1375
Maximal contact pressure (MPa)	49.7	60.9

**Table 3:** Maximum CNCs dispersion capability of different surfactants.

Surfactant (2%)	CNCs Max Con. (wt.%)
SDS	0.4
CTAB	0.3
SL 70	1.0
CAS	0.4
